# Eculizumab treatment in the salvage therapy of MOGAD: a case report

**DOI:** 10.3389/fimmu.2026.1861880

**Published:** 2026-07-27

**Authors:** Yuzhou Guo, Meiling Liu, Zhelin Zhang

**Affiliations:** Department of Neurology, Affiliated Hospital of Inner Mongolia Medical University, Hohhot, China

**Keywords:** case report, eculizumab, Epstein-Barr virus, MOGAD, ofatumumab

## Abstract

Myelin oligodendrocyte glycoprotein antibody-associated disease (MOGAD) is a rare inflammatory demyelinating disorder of the central nervous system. During the acute phase, management typically prioritizes high-dose methylprednisolone pulse therapy. When the patients show inadequate response, intravenous immunoglobulin (IVIG) or plasma exchange is recommended. We report the case of a 17-year-old male of Mongolian ethnicity who developed severe visual impairment following treatment for Epstein-Barr virus (EBV) meningitis, ultimately diagnosed with MOGAD. Following both methylprednisolone pulse therapy and IVIG, the patient exhibited a suboptimal response. However, his symptoms improved markedly after treatment with eculizumab. Subsequently, sequential therapy with ofatumumab was applied. At the six-month follow-up, the patient remained relapse-free and reported no adverse effects. This case suggests that eculizumab can serve as an effective salvage therapy for acute MOGAD refractory to conventional treatment. Ofatumumab may be a viable option for maintenance therapy during remission. These findings may provide valuable insights for future clinical research.

## 1 Introduction

Myelin oligodendrocyte glycoprotein (MOG) is widely expressed on the membrane of oligodendrocytes within the central nervous system. MOG antibody-associated disease (MOGAD) is a rare inflammatory demyelinating disorder of the central nervous system (1, 2). Although most patients respond to high-dose corticosteroids or intravenous immunoglobulin (IVIG) during the acute phase, a subset of patients, particularly those with MOGAD-associated optic neuritis, show suboptimal recovery or remain refractory to first-line therapies (3, 4). Complement-mediated damage has been implicated in the pathophysiology of MOGAD, raising the possibility that C5 inhibition might be therapeutically beneficial (5). However, to date, no pharmacological agent has been specifically approved for MOGAD, and the use of complement inhibitors in the acute setting remains largely unexplored. We report a case of Epstein-Barr virus (EBV) associated MOGAD. The patient exhibited an insufficient response to both methylprednisolone pulse therapy and immunoglobulin treatment; however, marked clinical improvement was observed following administration of eculizumab. The case is described in detail below.

## 2 Case presentation

The patient is a 17-year-old male of Mongolian ethnicity from Ulaanbaatar, Mongolia. He was a high school student at the time of presentation. The first admission occurred in May 2025, and the second (readmission) occurred in June 2025. He had no significant past medical history, including no history of hypertension, diabetes mellitus, immune system disorders, or other chronic diseases. He denied any history of smoking or alcohol use. Family history was negative for hereditary or genetic disorders, as well as autoimmune diseases.

**Figure 1:**
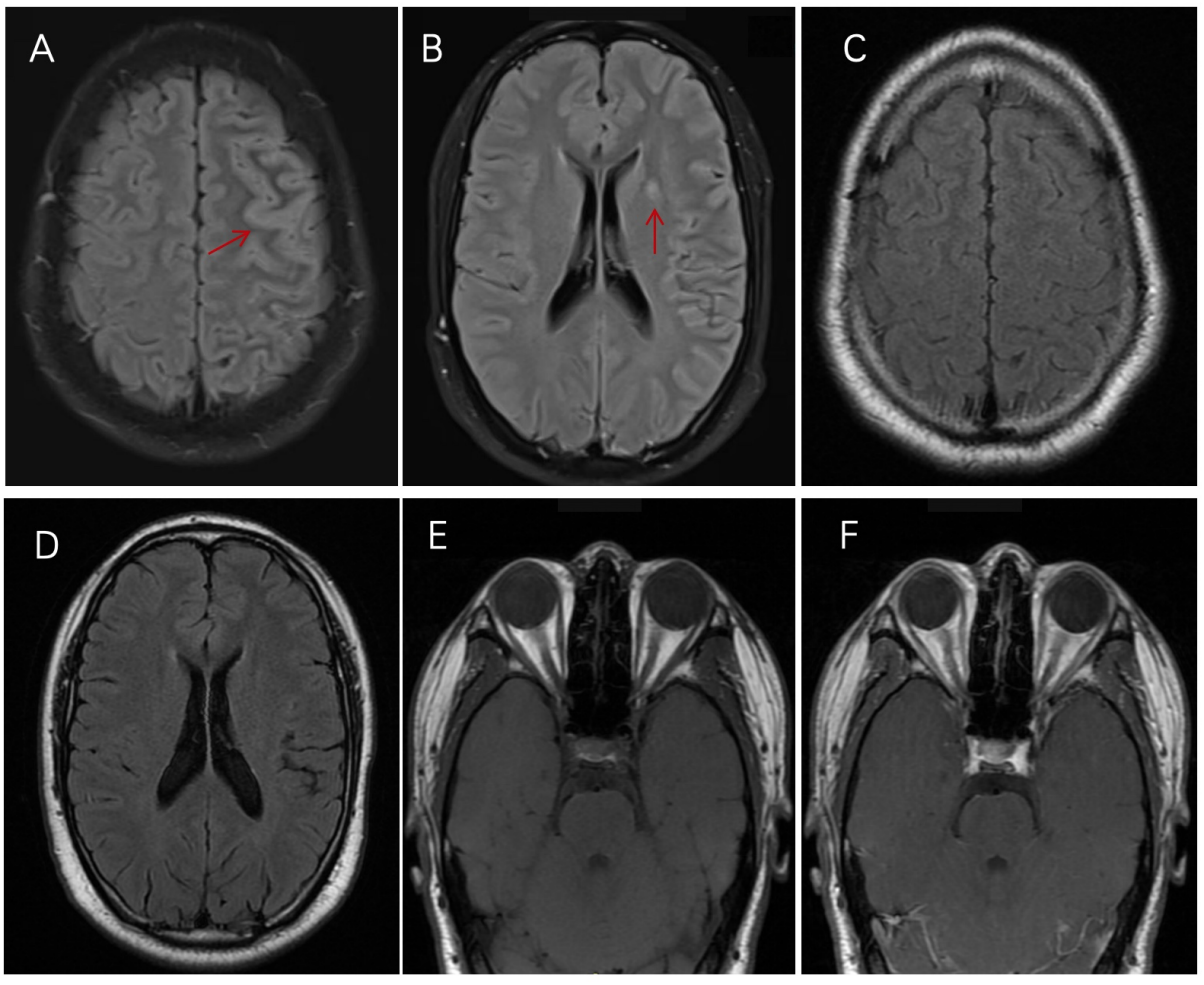
Magnetic resonance imaging. **(A)** Swelling of the left cerebral gyrus on FLAIR. **(B)** Hypersignal adjacent to the left lateral ventricle on FLAIR. **(C, D)** After antiviral treatment, the swelling of the gyri and the hyperintensity in the white matter on FLAIR sequences have both returned to normal. **(E, F)** No abnormalities of the optic nerve were observed on both T1 and T1-enhanced sequences.

**Figure 2:**
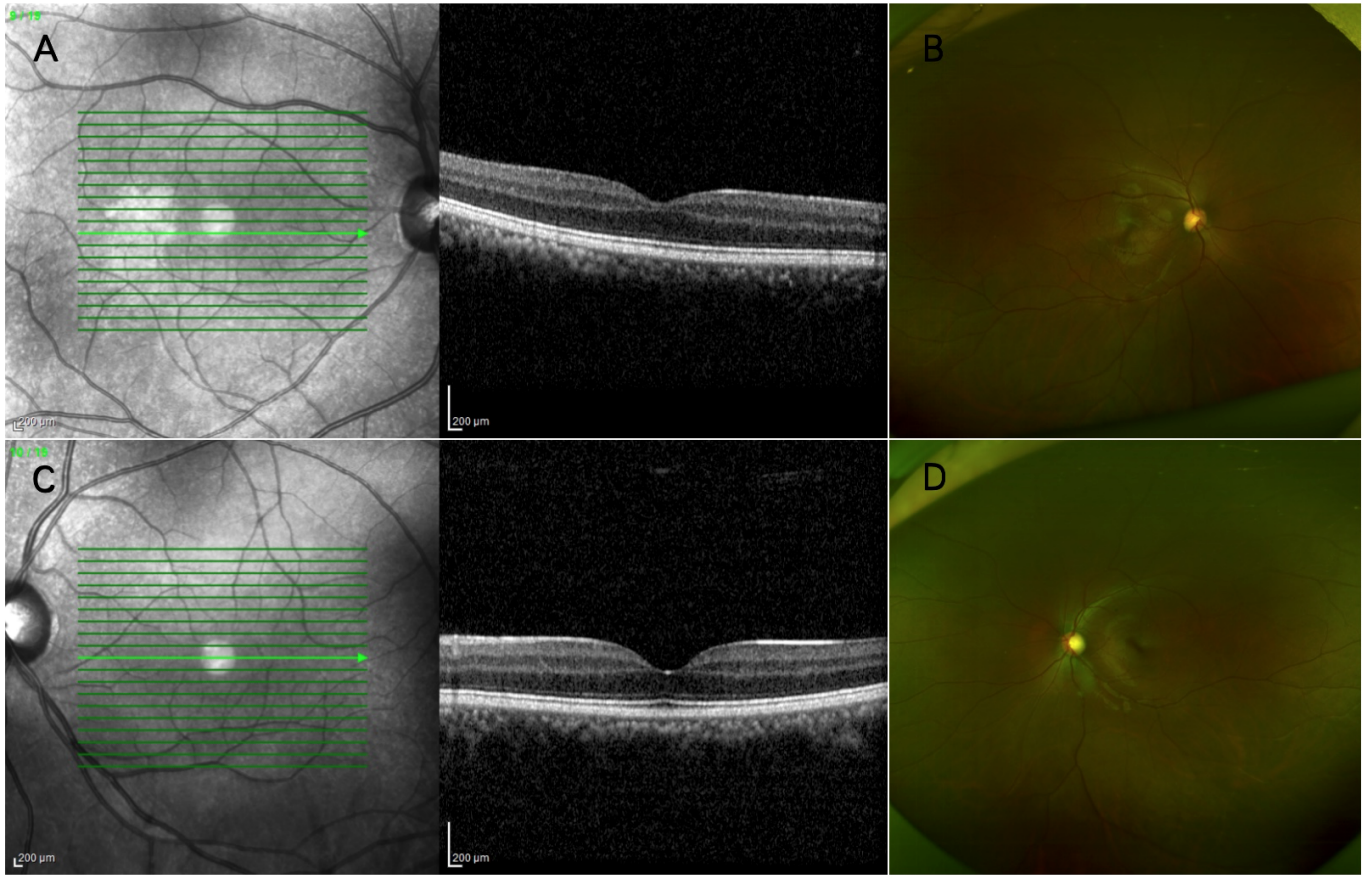
Ophthalmic examination. **(A)** Optical coherence tomography (OCT) of the right eye revealed no significant abnormalities. **(B)** Fundus photography of the right eye revealed no significant abnormalities. **(C)** OCT of the left eye revealed no significant abnormalities. **(D)** Fundus photography of the left eye revealed no significant abnormalities.

**Figure 3:**
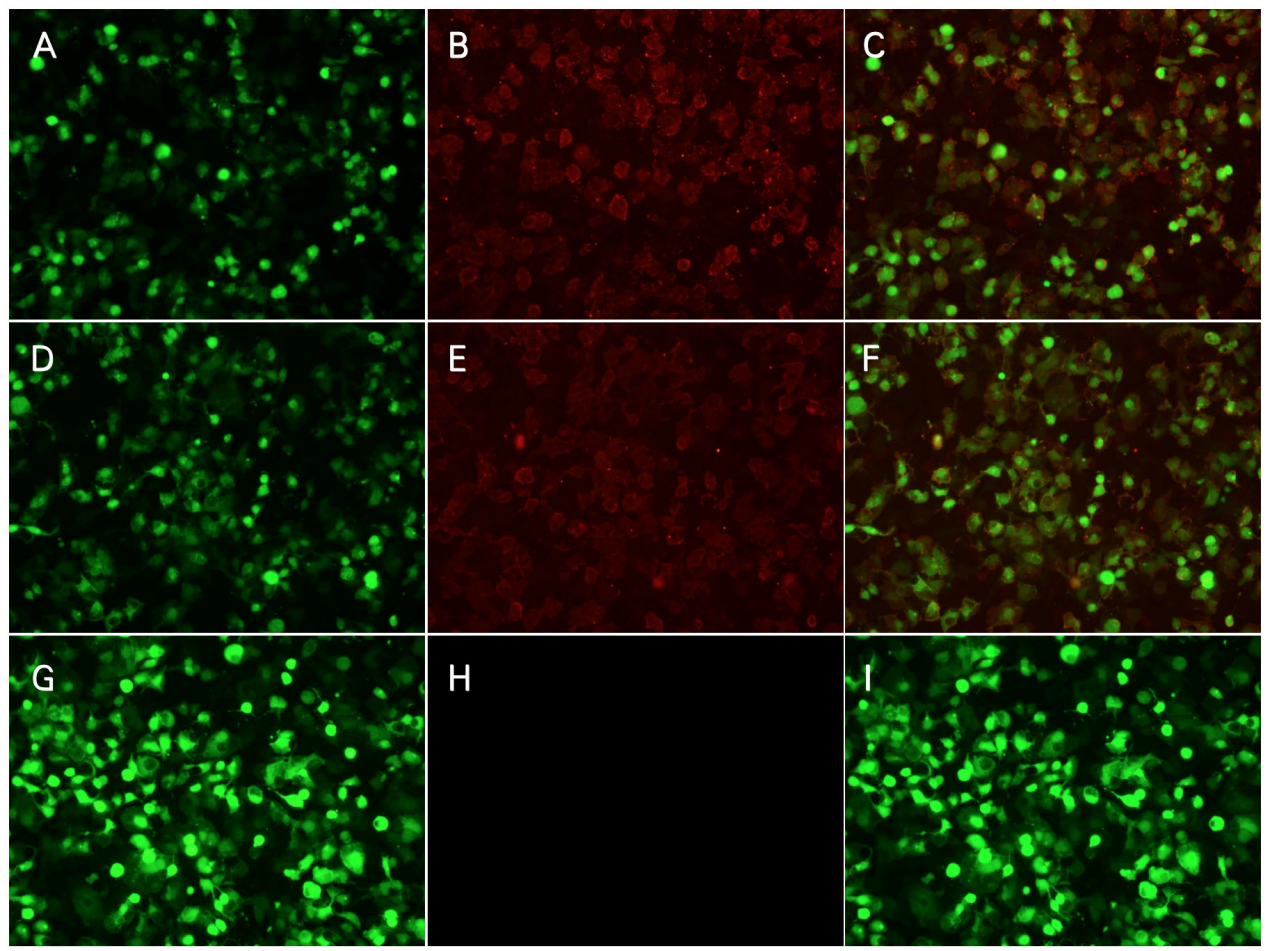
Dynamic changes of MOG antibodies. **(A)** Green fluorescence indicates that cells were successfully transfected with a plasmid co-expressing the MOG antigen and monomeric enhanced green fluorescent protein (EGFP), thereby confirming MOG antigen expression. **(B)** Red fluorescence illustrates the expression of MOG-IgG in the patient serum. **(C)** Colocalization of red and green fluorescence in specific cellular regions indicates a positive result for serum MOG-IgG. **(D–F)** CSF also tested positive for MOG-IgG. **(G–I)** Serum MOG-IgG became negative after treatment.

He was admitted with a chief complaint of fever and headache for 42 days, accompanied by decreased vision in both eyes for 1 week. Forty-two days prior to admission, the patient developed a headache associated with dizziness and vertigo, with additional nausea and vomiting. During the course of the illness, he experienced recurrent chills and high fever, with a maximum temperature of 39 °C. No seizures or motor deficits were reported. Laboratory investigations revealed a white blood cell count of 23.5 × 10^9^/L, a neutrophil percentage of 89%, and a C-reactive protein level of 15.25 mg/L. A further lumbar puncture demonstrated an opening pressure of 130 mm H_2_O and a cerebrospinal fluid (CSF) cell count of 448/mm³, with 61% neutrophils and 34% lymphocytes. Metagenomic next-generation sequencing of the CSF identified EBV infection (316 sequence reads). Cranial magnetic resonance imaging (MRI) showed swelling of the left frontal-parietal gyri and a demyelinating lesion adjacent to the left lateral ventricle (see **Figure 1A, B**). Neurological examination revealed nuchal rigidity and a positive Kernig’s sign. Accordingly, a diagnosis of EBV meningitis was established. The patient received antiviral therapy with acyclovir for 21 days and antibacterial therapy with meropenem for 14 days. His symptoms subsequently resolved, and he was discharged with a normalized body temperature. One week prior to readmission, the patient showed progressive bilateral blurred vision, which was more pronounced in the right eye. The initially mild blurred vision rapidly worsened. Three days prior to readmission, he experienced complete vision loss in the right eye and a darkened visual field, along with mild blurring in the left eye. No fever, headache, nausea, vomiting, or motor deficits were noted this time. On readmission, the patient received a physical examination, which revealed markedly reduced visual acuity in the right eye, limited to hand motion at 10 cm, while visual acuity in the left eye was relatively preserved. Meningeal irritation signs were negative. A follow-up brain MRI showed no significant abnormalities, with the swollen gyri returning to normal and the demyelinating lesions resolved (see **Figures 1C, D**). Contrast-enhanced MRI of the optic nerves showed no significant abnormalities (see **Figures 1E, F**). Optical coherence tomography (OCT) and fundus photography revealed no notable findings (see **Figure 2**). A repeated lumbar puncture revealed a CSF cell count of 21/mm³, with 99% mononuclear cells and 1% polymorphonuclear cells. Simultaneously, cerebrospinal fluid (CSF) and serum antibodies against MOG, aquaporin-4 (AQP4), and glial fibrillary acidic protein (GFAP) were detected using a cell-based assay (CBA). The results demonstrated that serum MOG antibodies were positive at a titer of 1:100+ (see **Figures 3A–C**), while CSF MOG antibodies were positive at a titer of 1:10+ (see **Figures 3D–F**). In contrast, both serum and CSF AQP4-IgG were negative, and GFAP antibodies were also negative. CSF oligoclonal bands were also negative.

Accordingly, a diagnosis of MOGAD was established based on the following diagnostic reasoning. The patient presented with a history of prodromal infection and acute onset of right-dominant bilateral optic neuritis. Serum MOG-IgG tested positive at a titer greater than 1:100, and CSF MOG-IgG was also positive at a titer greater than 1:10. In contrast, both serum and CSF AQP4-IgG were negative, effectively ruling out neuromyelitis optica spectrum disorder (NMOSD). Furthermore, CSF oligoclonal bands were negative, arguing against the diagnosis of multiple sclerosis (MS). Taken together, these findings led to the definitive diagnosis of MOGAD.

The patient received intravenous immunoglobulin therapy for 5 days, followed by a pulse therapy of methylprednisolone (1 g/day for 3 days, then 500 mg/day for 3 days). By the end of the first week of treatment, the patient exhibited clinical improvement: visual acuity in the left eye had largely returned to normal, whereas the right eye was limited to hand motion at a distance of 3 m. Formal visual acuity testing demonstrated 0.05 in the right eye and 1.0 in the left eye. During subsequent tapering of methylprednisolone from 500 mg to 120 mg, the improvement in the patient’s vision plateaued. Repeat serum testing showed a persistent MOG antibody titer of 1:100+. To break through this therapeutic bottleneck and facilitate further visual recovery, plasma exchange was considered. However, given that only 11 days had elapsed since the last immunoglobulin dose and that plasma exchange is invasive, a careful assessment of the associated risks and benefits was required. Consequently, it was decided to administer eculizumab, a C5 complement inhibitor that targets the downstream pathway of the autoimmune response. The patient received eculizumab treatment (900 mg) via weekly intravenous infusion, in combination with oral prednisone acetate (60 mg/day). Furthermore, to mitigate the risk of meningococcal infection associated with eculizumab therapy, a single dose of ceftriaxone (2 g) was administered intravenously prior to the initiation of eculizumab, followed by regular oral amoxicillin at a dosage of 500 mg twice daily. On the second day following the first eculizumab infusion, the patient reported gradual visual improvement. By day 6, right eye visual acuity had improved to 0.7. In the second week, treatment was continued with a second 900 mg dose of eculizumab and prednisone acetate (55 mg/day). By day 6 of the second treatment cycle, right eye visual acuity had further improved to 0.9, approaching normal levels, although the patient reported mild residual dyschromatopsia.

Over the subsequent two weeks, the patient received two additional doses of eculizumab, with gradual tapering of prednisone. Following the fourth eculizumab infusion, visual acuity in both eyes reached 1.0, as confirmed by formal testing. The patient reported high satisfaction with the visual recovery. He successfully participated in the Chinese National College Entrance Examination (Gaokao) and was subsequently admitted to a university in northern China, which he was attending at the time of this report. He noted that he was able to return to all normal daily activities, including reading, using electronic devices, and attending classes without difficulty. To prevent disease recurrence, maintenance therapy with ofatumumab was initiated, and prednisone was further tapered. The patient adhered fully to the ofatumumab regimen and all scheduled follow-up visits, with no missed doses or discontinuation of treatment. The treatment was well tolerated; no infusion-related reactions, infections, or other adverse events were observed during the follow-up period. Serum MOG antibodies became negative two months after initiation of ofatumumab therapy. (see **Figures 3G–I**).

**Figure 4:**
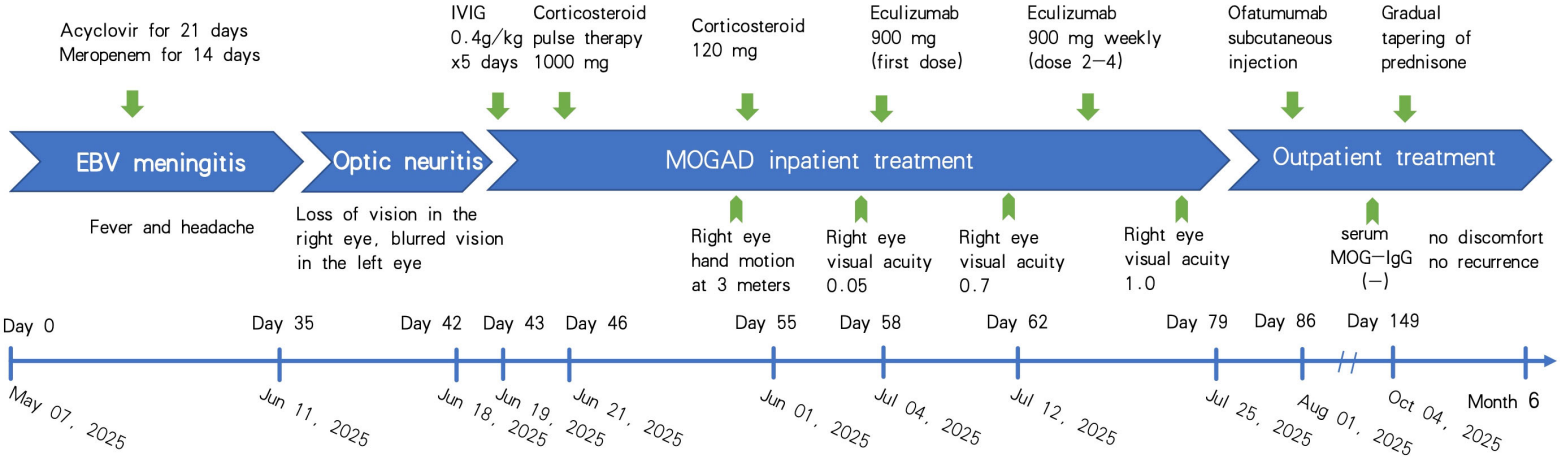
Clinical course.

At the six-month follow-up, visual acuity remained 1.0 in both eyes, with no reported discomfort, and serum MOG antibodies remained negative. The patient continues to be followed up regularly. The clinical course is illustrated in **Figure 4**.

## 3 Discussion

MOGAD is a rare autoimmune inflammatory demyelinating disorder of the central nervous system. Its typical clinical manifestations include optic neuritis, transverse myelitis, acute disseminated encephalomyelitis (ADEM), and brainstem encephalitis (1). Its estimated prevalence ranges from 0.51 to 3.7 per 100,000 individuals (2). In this present case, we reported a patient who exhibited a suboptimal response to conventional therapy with corticosteroids combined with intravenous immunoglobulin. However, following administration of eculizumab, his clinical symptoms improved rapidly and ultimately resolved completely. Previous studies indicate that approximately 60% of patients with MOGAD have a history of preceding infection. Viral infections may contribute to the production of MOG-IgG or trigger disease onset. EBV has long been implicated in the pathogenesis of MS, although its association with MOGAD remains controversial (6). Nevertheless, several case reports have described the occurrence of MOGAD following EBV infection (7–9). In addition, a Japanese observational study demonstrated a significant association between EBV infection and the onset of both MS and MOGAD (10). In the present case, a clear temporal association was observed between EBV infection and the subsequent onset of MOGAD. This association suggests a potential link between the two events; however, further validation is required. At present, this observation supports only the hypothesis that EBV infection may serve as a triggering factor for MOGAD.

Patients with MOGAD may exhibit clinical improvement with therapies such as corticosteroids, intravenous immunoglobulin (IVIG), and rituximab. However, no pharmacological agents have yet been globally approved specifically for the treatment of MOGAD. Universally, it is believed that during the acute phase, patients are generally considered responsive to intravenous methylprednisolone (3). Conversely, observational studies indicate that following acute-phase therapy, up to 47% of patients with MOGAD-associated optic neuritis experience suboptimal outcomes or only partial remission. For patients who are unresponsive to corticosteroids, IVIG, or plasma exchange, it is often associated with improved outcomes (4). In the present case, the patient exhibited limited clinical improvement following combined corticosteroid pulse therapy and IVIG. Moreover, dynamic monitoring of serum MOG antibody titers demonstrated no significant decline after these treatments. This may suggest persistent inflammatory activity and ongoing complement activation and autoantibody-mediated responses, which hinder remyelination and functional recovery. Following the administration of eculizumab, a C5 complement inhibitor, the patient’s symptoms improved markedly. Eculizumab is a humanized monoclonal antibody that prevents the cleavage of complement component C5 into the proinflammatory fragments C5a and C5b. This antibody has been reported to significantly reduce relapse risk in patients with aquaporin-4 (AQP4)-IgG-positive NMOSD (11, 12). Currently, eculizumab is considered a first-line therapy for relapse prevention in this population. In MOGAD, MOG-IgG autoantibodies produced by B cells bind to MOG, activating the complement system and leading to the formation of membrane attack complexes. In addition, these antibodies contribute to the immune response cascade through antibody-dependent cellular cytotoxicity and T-cell-mediated inflammatory responses, ultimately resulting in demyelination and oligodendrocyte death (13). Experimental studies in murine models suggest that approximately half of MOG antibody-mediated demyelination is mediated via Fc receptor activation, while the other half is complement-dependent (14). Keller et al. compared the serum complement levels in patients with MOGAD, NMOSD, MS, and in healthy controls, revealing significantly elevated levels of both classical and alternative complement pathway activation products in patients with MOGAD and suggesting that complement activation is a key pathological feature of this disease (5). MOG-IgG-mediated activation of the complement cascade promotes inflammation and membrane attack complex formation, thereby contributing to neuronal and myelin injury (15, 16). To our knowledge, this is the first clinical report demonstrating the successful use of eculizumab as an acute rescue therapy for a patient with MOGAD refractory to conventional first-line treatments. The comprehensive longitudinal follow-up, including serial MOG antibody titers and detailed visual acuity assessments, provides robust evidence of the temporal association between eculizumab administration and clinical improvement. Furthermore, the patient’s complete seroconversion (MOG antibody negativity) and sustained functional recovery (return to normal schooling and university admission) offer objective support for the potential efficacy of this approach.

Approximately 50%-60% of patients with MOGAD experience relapse. B-cell-depleting therapies, oral immunosuppressants, and interleukin-6 receptor inhibitors are commonly used as sequential therapeutic treatments during remission (17). However, an international multicenter retrospective study involving 121 patients with MOGAD demonstrated that, although rituximab reduced the risk of relapse by approximately 37%, the 1-year relapse-free rate was 55%, which additionally declined to 33% at 2 years (18). These findings indicate that rituximab is less effective in preventing relapse in MOGAD than in NMOSD (19). Previous research has indicated that more than one-third of patients experience relapse despite complete B-cell depletion during rituximab therapy (19). In the present case, given the disability of initial visual impairment, ofatumumab treatment was selected to prevent relapse. The patient remained relapse-free during the six-month follow-up period. Ofatumumab not only induces B-cell depletion but may also promote negative conversion of MOG antibodies. Similarly, Chen et al. (6) reported two cases of EBV-induced MOGAD in which favorable outcomes were achieved with ofatumumab combined with methylprednisolone. Taken together, these findings suggest that ofatumumab may represent a promising option for relapse prevention in MOGAD.

Our report has the following limitations. First, this study is a single case report. Eculizumab was administered following high-dose glucocorticoid pulse therapy combined with intravenous immunoglobulin. Therefore, it is difficult to definitively distinguish whether the patient’s visual recovery was attributable to the therapeutic effect of eculizumab or to the delayed subsequent effects of the high-dose corticosteroids and immunoglobulin. This phenomenon should be interpreted with caution, and future clinical studies are required to validate the precise role of eculizumab in the acute-phase treatment of patients with MOGAD. Second, the observation period for this case was relatively short. Although the patient’s MOG antibodies became negative, the recurrence of MOGAD remains unpredictable. Consequently, long-term follow-up is necessary to verify the preventive effect of ofatumumab in this patient. Similarly, more rigorous clinical studies are needed to clarify the overall efficacy of ofatumumab in preventing MOGAD relapses.

## 4 Patient perspective

The patient reported marked visual improvement beginning on the second day after the first eculizumab infusion. By the end of week one, his right eye vision had recovered sufficiently for normal schooling and daily activities. He expressed high satisfaction with the treatment and experienced no significant adverse effects, although he noted the inconvenience of weekly hospital visits.

## 5 Conclusion

Overall, we report a case of EBV-induced MOGAD. Here, conventional acute-phase therapies, including high-dose methylprednisolone pulse therapy and immunoglobulin, appeared insufficient. Following rescue therapy with eculizumab, marked clinical improvement was observed. This single case suggests that eculizumab might represent a potential rescue option for acute MOGAD refractory to standard treatment, although further experience is needed. In addition, ofatumumab could be considered as a possible alternative for preventing disease recurrence, pending future investigation.

## Data Availability

The original contributions presented in the study are included in the article/supplementary material. Further inquiries can be directed to the corresponding author.
